# Sutureless and Rapid Deployment Prosthesis in Redo-Bentall Endocarditis

**DOI:** 10.1155/2024/9455342

**Published:** 2024-10-16

**Authors:** Salvatore Nicolardi, Gabriele De Masi De Luca, Federica Mangia, Cosimo Angelo Greco, Salvatore Zaccaria

**Affiliations:** ^1^Department of Cardiac Surgery, “Vito Fazzi” Hospital, Lecce, Italy; ^2^Cardiology Department, “Card. Panico” Hospital, Tricase (Le), Italy; ^3^Department of Life, Health and Environmental Sciences, University of L'Aquila, L'Aquila, Italy

## Abstract

Aortic valve replacement (AVR) in a patient with a bio-Bentall conduit can be very challenging, especially if there was a previous endocarditis process for significant morbidity and mortality. We report a case of sutureless AVR in an old patient with a bio-Bentall conduit (Carpentier–Edwards Perimount Magna Ease 25 aortic valve and Hemashield 30 aortic conduit), who developed an endocarditis on aortic prosthesis valve. We believe that sutureless AVR is the best option for redo-operation in older patients with a high surgical risk because it allows for easy rapid deployment implantation, avoids anchoring sutures on a fragile aortic anulus, and reduces cardiopulmonary and aortic cross-clamp times. In this setting, it should be considered as a safe and valid alternative not only to traditional prosthesis but also in selected cases to transcatheter valve-in-valve solutions.

## 1. Case Report

A 77-year-old man was referred to our center because of endocarditis on Carpentier–Edwards 25 aortic bioprosthesis in previous Bentall-De Bono modified operation and coronary artery bypass grafting (left internal mammary artery on anterior descending coronary artery and venous bypass graft on obtuse marginal). Cardiac surgery had been performed 5 years earlier for aneurysm of the aortic root and ascending aorta in bicuspid valve and coronary heart disease. The diagnosis of blood culture negative infective endocarditis (BCNIE) was performed in a nearby spoke hospital cardiology center, where empiric antibiotic therapy had been started. The patient's comorbidities included hypertension, previous smoking, chronic obstructive pulmonary disease, ictus cerebri without neurological relics, monoclonal gammopathy of undetermined significance, and prostatectomy for adenocarcinoma. A history of recent persistent fever was referred, but no specific event that could have oriented the origin of the fever is reported.

Transthoracic echocardiography showed a 17-mm vegetation attached to the suture annulus engaging in diastole left ventricular outflow and resulting in severe aortic bioprosthesis regurgitation (pressure half time <200 ms). Transesophageal echocardiography examination confirms the absence of perivalvular abscess or fistula.

During hospitalization, the patient underwent grafts and coronary angiography that showed patency of the left internal mammary artery on the anterior descending coronary artery and of the venous bypass graft on obtuse marginal: only nonstenosing atheromatous plaques on the right coronary artery. Blood cultures were negative. The calculated logistic EuroSCORE II was 34.35%, and the society of thoracic surgeons (STS) calculated risk of mortality was 36.778%. Thoracic approach was performed through previous median sternotomy. After tenacious connective adhesions have been lysed with electric scalpel, aortic cannulation in the distal portion of the ascending aorta and right atrial cannulation for cardiopulmonary bypass (CPB) were performed. The left ventricle was vented through the upper right pulmonary vein. Core body temperature was dropped to 35°C and the myocardium protected by an anterograde infusion of Custodiol cardioplegia through selective cannulation of the coronary ostia. Left internal mammary artery (LIMA) grafted on anterior interventricular artery (IVA) was previously clamped with bulldog before starting the cardioplegia infusion.

Carbon dioxide CO_2_ in the surgical field was used to reduce the risk of air embolism. After cross-clamp, sutureless implantation valve required 1.5–2 cm higher incision of Hemashield Dacron tubular prosthesis than a standard aortotomy for stented valves.

**Figure 1:**
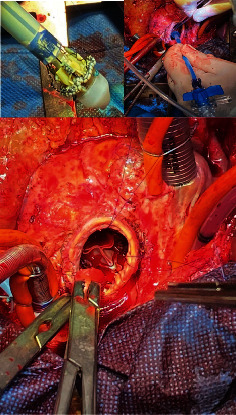
Sutureless and rapid deployment Perceval S implantation phases.

Traction sutures on the aortic Dacron wall improved exposure of degenerated bioprosthesis, which had an endocarditis vegetation attached to the suture ring. The bioprosthesis was excised and removed together with the vegetation. The anatomical annulus did not present any abscess. It was thoroughly surgical reclaimed and sized with appropriate valve sizers. Afterward, three guiding 4-0 Prolene sutures were placed at the nadir point of each valve sinus for an accurate alignment of the inflow portion of the prosthesis into the aortic annulus. The valve was collapsed using a specific device system and connected to the guiding sutures. Then it was parachuted down into the aortic annulus and released. The size of the Perceval prosthesis is small (S) (Figure 1).

**Figure 2:**
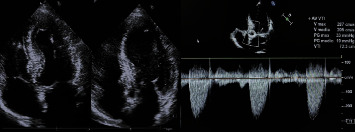
Echocardiographic evaluation at 12 months.

Finally, the aortotomy was closed using 4-0 running sutures. CPB and aortic cross-clamp (ACC) times were 78 and 69 min, respectively. The patient was extubated 7 h after surgery and was held in the intensive care unit (ICU) for 3 days. Biocultures of the aortic prosthesis exported with the endocarditis vegetation were subsequently negative, but considering the persistency of high inflammatory marker levels, empiric antibiotic therapy was continued for 6 weeks. Correct implantation of the prosthesis was confirmed with intraoperative transesophageal echocardiography. The patient experienced an uneventful postoperative course and was discharged on the 10th day postoperatively. We have obtained an excellent result even in the immediate postoperative period through fast-tracking the patient and facilitating early discharge. The patient immediately resumed a normal life, and echocardiographic follow-up at 12 months demonstrated a correct positioning and functioning of the aortic sutureless bioprosthesis (Figure 2).

## 2. Discussion

Management of degenerated bioprosthetic aortic valves remains a challenge. Valve-in-valve transcatheter aortic valve replacement (AVR) has limited utility in the presence of small annuli/prosthetic valves. Sutureless valves may offer an advantage over traditional redo AVR by maximizing effective orifice area due to their unique design as well as ease of implant [1]. In recent years, several experiences have been described that demonstrate the effectiveness of sutureless and rapid deployment prosthesis implantation in redo-operations [2–4] also in endocarditic bioprosthetis degeneration [5–6]. Our case report confirms the benefits of this surgical approach in patients with redo-Bental endocarditis. It is a simple and reproducible technique to replace a degenerated bioprosthetis by endocarditis while preserving the previous aortic root replacement. However, it is important to keep in mind that there are no abscesses at the level of the annulus. The use of sutureless and rapid deployment prosthesis reduces the time of CPB and ACC, increasing the chances of surgical success and patient survival and helping reoperated patients to a rapid functional recovery. If long-term durability is confirmed, sutureless valves should be considered in a broader population of patients always considered to be at high risk and with a high perioperative mortality rate.

## Data Availability

The data that support the findings of this study are available from the corresponding author upon reasonable request.
